# Preparing practical in-person evidence-based journal club in COVID-19 crisis

**DOI:** 10.30476/jamp.2020.86217.1225

**Published:** 2020-07

**Authors:** SEYYED SAEED KHABIRI

**Affiliations:** 1 Department of orthopedic surgery, Faculty of medicine, Kermanshah University of Medical sciences, Kermanshah, Iran

**Dear Editor**,

During the Corona virus epidemic, most jobs closed, and according to the policies to reduce crowding, most educational sessions such as clinical rounds, morning reports, and journal clubs barred; therefore, it is necessary to consider ways to deal with these limitations. 

The use of virtual training used to be limited due to the distance between people, but during the Corona-virus era, there was an opportunity to use this technique more ( 1 ). One of the chapters in residency training is the Journal club, which plays an important role in raising the level of knowledge and decision-making power, and using the experiences of others in medical performance ( 2 ).

Nowadays, evidence-based medicine (EBM) is an accepted educational paradigm at all levels of medical education ( 3 ). EBM relies on  accountability, and clear and intelligent use of the current best evidence in developing management about the care of particular patients ( 4 ). There are four mainstay of EBM: building up a question, searching the literature, appointing the relevant article, and performing critical appraisal. Therefore, in this article, we aimed to properly provide a practical model for presenting the evidence-based journal club, which is also possible in online or in-person programs.

In this model, unlike journal club session, which starts with an interesting article or a popular journal, an EBM journal club starts with a patient’s problem. In this case, our recommendation is that a mentor and two residents (senior and junior) should identify a patient whose condition is mostly traumatic or emergency due to the closure of elective surgery.

Generate a clinical question based on the PICO (patient, intervention, comparison and outcome) model. This question must be certain, well-structured and answerable ( 5 ). At that time, under the supervision of a mentor, the search is undertaken in the databases using search engines to finally provoke one or more related articles.

Up to now, only three people have been involved. But at this point, the PICO question and the articles obtained are introduced to other members (other faculty members and residents) in the virtual groups from 3 days before the program, so that the people present at the meeting know about them.

On the day of the journal club, the time of which have already been defined, mentors and residents will take control of the meeting. At the beginning, the junior resident explains the clinical question and the how made PICO query and then how to search (about 5 minutes) and the senior resident describes critical appraisal articles (about 10 minutes) which have different items depending on the type of article.

Next, after the end of the critiques, the mentor respectfully asks the faculty members and summarizes the excellent results of the articles with the real working environment and the attendance opinions.

Our recommendation is that the PICO question, the search method, the articles obtained, the critique of the articles, and the comments of the attendees should be written, properly recorded in a special form attached, and be published on certain sites, such as https://digitalcommons.wayne.edu/crp/.

Finally, these days, when there are limitations for the academic community, virtual learning capacities can be properly used in groups.

*Take home message:*

In the social constraints of covid-19, it is possible to use virtual communication platforms to make journal club session based on EBM principles in a completely dynamic and productive way, and to continue education at all levels, despite this world-wide crisis.
